# Supplementary material for: Giant piezoelectric voltage coefficient in grain-oriented modified PbTiO3 material
Source: Nat Commun. 2016 Oct 11;7:13089. doi: 10.1038/ncomms13089 (PMC5062610; doi:10.1038/ncomms13089)
Supplement: Supplementary Information — Supplementary Figures 1-4, Supplementary Notes 1-2 and Supplementary References. [file ncomms13089-s1.pdf]

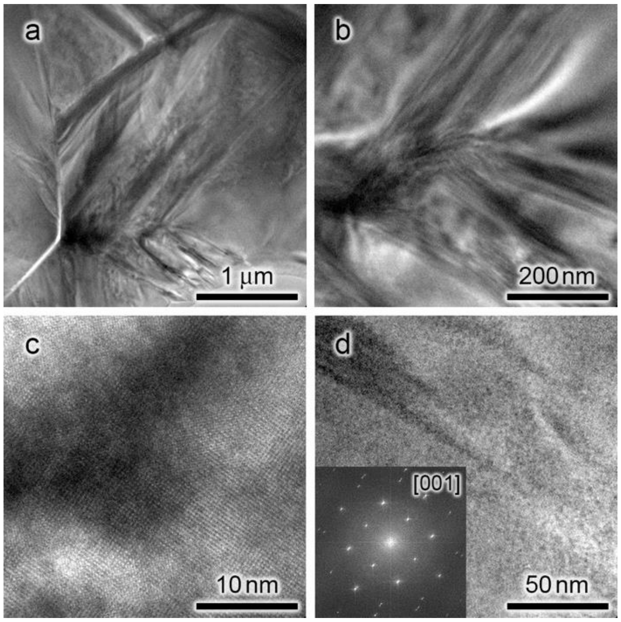

Supplementary Figure 1 | Domain structures of textured SM-PT ceramic under TEM. (a) Bright field TEM image of the ferroelectric domains in textured PT. (b) Magnified view of the intersection of the 90° domains. (c) HR-TEM images with strain contrast at the intersection of the 90° domains. (d) High resolution image of the edge of the domain wall. The inset of (d) shows fast Fourier transform (FFT) of the corresponding image.

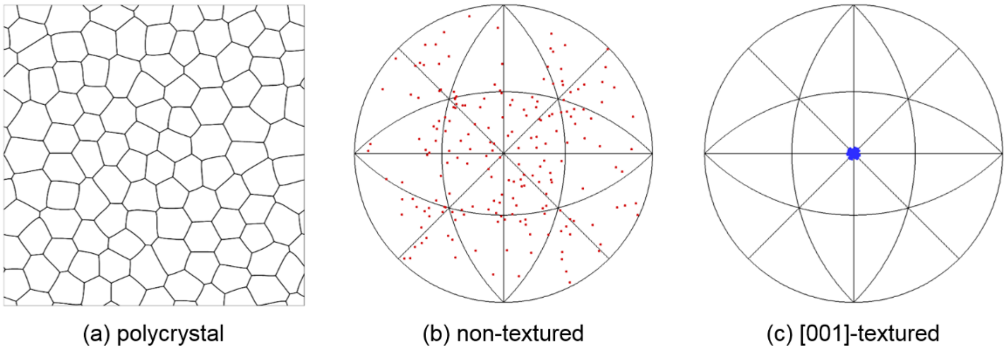

Supplementary Figure 2 | Phase field model of ferroelectric ceramics. (a) Grain structure of a polycrystal used to model the ferroelectric ceramic sample. Stereographic projection of (001) plane orientation distribution in (b) non-textured and (c) [001]-textured polycrystals.

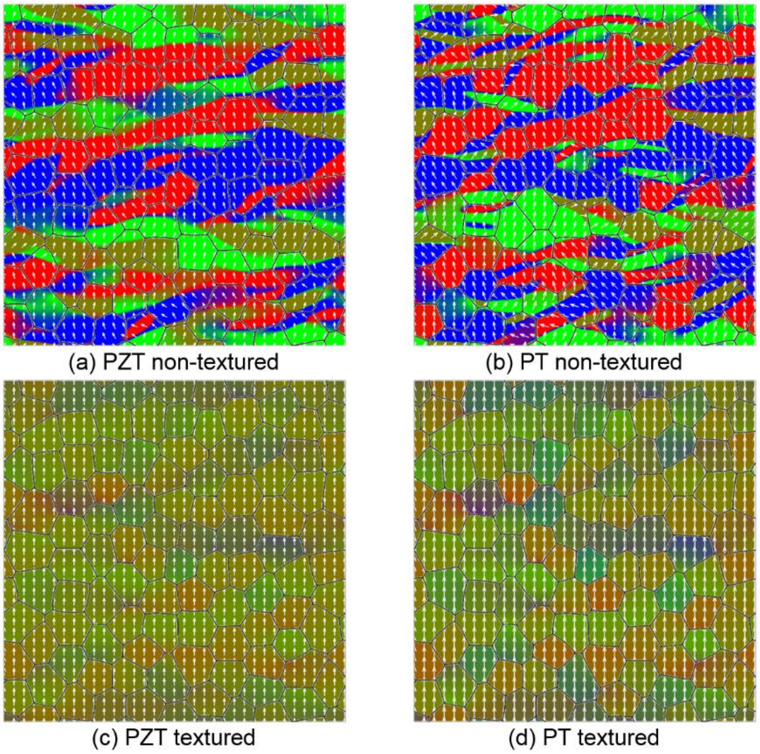

Supplementary Figure 3 | Phase field simulation of polarization distribution and domain structures in poled ferroelectric ceramics. (a) non-textured PZT, (b) non-textured PT, (c) [001]-textured PZT, and (d) [001]-textured PT. White arrows represent local polarization vectors. Colors depict the two polarization components deviating from the poling (vertical) direction. The simulation system is discretized into 512×512 computational grids with periodic boundary conditions.

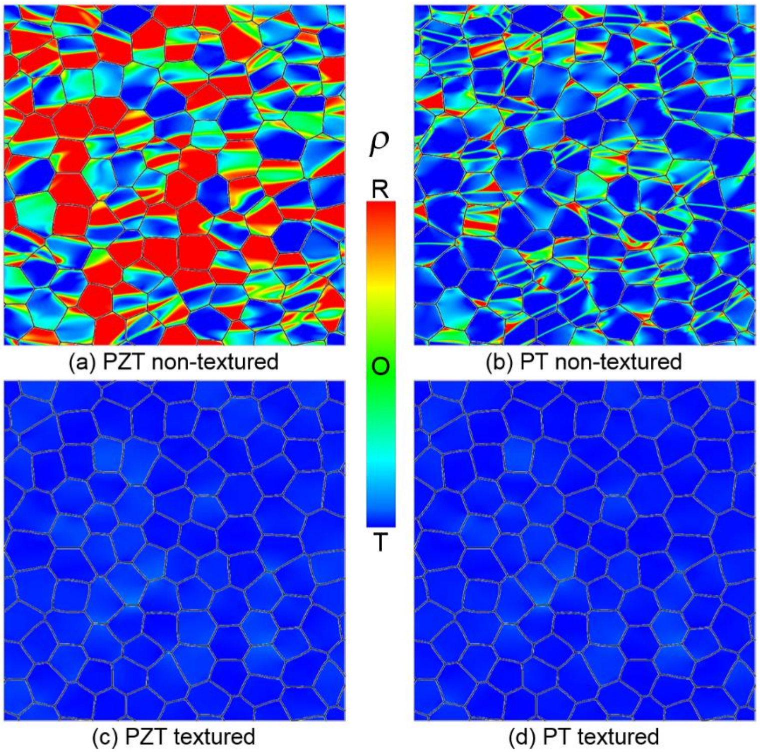

Supplementary Figure 4 | Phase field simulation of phase morphology in poled ferroelectric ceramics. (a) non-textured PZT, (b) non-textured PT, (c) [001]-textured PZT, and (d) [001]-textured PT. Colors are coded by parameter $\rho = \left(|P_1P_2| + |P_2P_3| + |P_3P_1|\right)/P^2$ in terms of polarization vector $\mathbf{P}(\mathbf{r})$ in local lattice coordinate system of individual grains and depict tetragonal (T, $\rho = 0$, blue), orthorhombic (O, $\rho = 0.5$, green) and rhombohedral (R, $\rho = 1$, red) phases. The simulation system is discretized into 512×512 computational grids with periodic boundary conditions.

Supplementary Note 1: Domain structures of textured SM-PT ceramic under TEM

Supplementary Figure 1a-c show bright field TEM images for the textured specimen, revealing lamellar 90° domains with planar $\{100\}_C$ domain walls. The electron beam was parallel to the $[001]_c$ orientation. In this figure, one can clearly observe larger 90° domain morphology with width of about 200-500 nm. These wide domains are difficult to switch due to higher activation energy. The magnified view of the intersection of these 90° domains is shown in **Supplementary** Figure 1b. The plane of intersection of these 90° domains is $\{110\}_C$ resulting in herringbone-type morphology. The magnified view of the domain intersection revealed presence of substructures inside the macrodomains. Considering the case of two lamellar domains with a common domain wall attracting each other, the force of attraction decreases rapidly with the increase in the distance between the two domain walls, resulting in fine substructure ranging from submicron to nano scale in dimension[1]. The strain contrast due to the intersection of the domains is shown in **Supplementary** Figure 1c. The magnified view of the morphology at the edge of a lamellar domain is shown in **Supplementary** Figure 1d. The splitting of the spots in the FFT pattern (inset of **Supplementary** Figure 1d) is due to domain wall. The interaction between domains walls leads to bending of the domain walls and other interboundary effect. The actual angle of the 90° domain wall can be given by $2tan^{-1}(a/c)$. In $BaTiO_3$, the actual angle of 90° walls is 89.4^o. However, the angle of domain wall is forced to be 90° instead of 89.4^o which results in localized strained regions whose strength varies with the grain size. The localized strained regions can also act as nucleation sites for domain switching. In the case of $PbTiO_3$, the higher tetragonality (c/a=1.06) results in the domain wall angle of 86.66^o. This local strain will influence the converse piezoelectric response.

Supplementary Note 2: Phase field model of ferroelectric polycrystals.

We employ the ferroelectric polycrystal model[2], which is capable of simulating realistic grain structures and textures. The state of a ferroelectric polycrystal is described by the polarization vector field $\mathbf{P}(\mathbf{r})$, and the total system free energy under externally applied electric field $\mathbf{E}^{\mathrm{ex}}$ is given as[3-6]:

$$F=\int d^3r\left[f\left(R_{ij}P_j\right)+\frac{\beta}{2}\frac{\partial P_i}{\partial r_j}\frac{\partial P_i}{\partial r_j}-E_k^{\mathrm{ex}}P_k\right]+\frac{1}{2}\int\frac{d^3k}{\left(2\pi\right)^3}\left[\frac{n_in_j}{\varepsilon_0}\tilde{P}_i\tilde{P}_j^*+K_{ijkl}\tilde{\varepsilon}_{ij}\tilde{\varepsilon}_{kl}^*\right]$$

(Supplementary Equation 1)

where

$$\begin{aligned}f\left(\mathbf{P}\right)&=\alpha_1\left(P_1^2+P_2^2+P_3^2\right)+\alpha_{11}\left(P_1^4+P_2^4+P_3^4\right)+\alpha_{12}\left(P_1^2P_2^2+P_2^2P_3^2+P_3^2P_1^2\right)\\&+\alpha_{111}\left(P_1^6+P_2^6+P_3^6\right)+\alpha_{112}\left[P_1^4\left(P_2^2+P_3^2\right)+P_2^4\left(P_3^2+P_1^2\right)+P_3^4\left(P_1^2+P_2^2\right)\right]\\&+\alpha_{123}P_1^2P_2^2P_3^2\end{aligned}$$

(Supplementary Equation 2)

is Landau-Ginzburg-Devonshire free energy function of ferroelectric single crystal[7]. It is worth noting that $\mathbf{P}(\mathbf{r})$ in Supplementary Equation 1 is defined in a global coordinate system attached to the polycrystal, while $\mathbf{P}(\mathbf{r})$ in Supplementary Equation 2 is defined in a local coordinate system aligned with <100> lattice axes of a ferroelectric single crystal, and the operation $R_{ij}P_j$ in $f\left(R_{ij}P_j\right)$ in Supplementary Equation 1 transforms $\mathbf{P}(\mathbf{r})$ from the global sample system to the local lattice system in each grain, where $\mathbf{R}(\mathbf{r})$ is a grain rotation matrix field that describes the geometry (shape, size, location) and crystallographic orientation (texture) of individual grains in the polycrystal[2]. Supplementary Figure 2a shows the structure of 100 grains in a polycrystal that is used in the simulation study, and Supplementary Figure 2b and Supplementary Figure 2c show the stereographic projection of (001) plane orientation distribution in non-textured and [001]-

textured polycrystals, respectively. The gradient term in Supplementary Equation 1 characterizes energy contribution from polarization gradient in domain wall regions. The **k**-space integral terms characterize the domain configuration-dependent energies of long-range electrostatic and elastostatic interactions, where ε_0 is permittivity of free space, $\tilde{\mathbf{P}}(\mathbf{k})$ and $\tilde{\boldsymbol{\varepsilon}}(\mathbf{k})$ are the Fourier transforms of the respective field variables $\mathbf{P}(\mathbf{r})$ and $\boldsymbol{\varepsilon}(\mathbf{r})$. The spontaneous strain $\boldsymbol{\varepsilon}$ is coupled to polarization $\mathbf{P}$ through electrostriction coefficient tensor Q_{ijkl}, $\varepsilon_{ij} = Q_{ijkl}P_kP_l$. $K_{ijkl}=C_{ijkl}-n_mC_{ijmn}\Omega_{np}C_{klpq}n_q$, $\Omega_{ik}=(C_{ijkl}n_jn_l)^{-1}$, C_{ijkl} is elastic modulus tensor, and $\mathbf{n}=\mathbf{k}/k$. The spatial-temporal evolution of the polarization $\mathbf{P}(\mathbf{r},t)$ in response to varying electric field $\mathbf{E}^{ex}(t)$ is characterized by the time-dependent Ginzburg-Landau equation[3-6]:

$$\frac{\partial \mathbf{P}(\mathbf{r},t)}{\partial t} = -L\frac{\delta F}{\delta \mathbf{P}(\mathbf{r},t)} \qquad \text{(Supplementary Equation 3)}$$

where L is the kinetic coefficient. In this work, we use the experimentally determined composition-dependent material parameters of $Pb(Zr_{1-x}Ti_x)O_3$ (PZT)[8,9].

Supplementary References

1. Salje, E., Kuscholke, B. & Wruck, B. Domain wall formation in minerals: I. Theory of twin boundary shapes in Na-feldspar, *Phys. Chem. Min.* **12**, 132-140 (1985).
2. Wang, Y. U. Field-induced inter-ferroelectric phase transformations and domain mechanisms in high-strain piezoelectric materials: insights from phase field modeling and simulation, *J. Mater. Sci.* **44**, 5225-5234 (2009).
3. Yang, W. & Chen, L. Q. Computer simulation of the dynamics of 180° ferroelectric domains, *J. Am. Ceram. Soc.* **78**, 2554-2556 (1995).

4. Hu, H. L. & Chen, L. Q. Computer simulation of 90° ferroelectric domain formation in two-dimensions, *Mater. Sci. Eng. A* **238**, 182-191 (1997).

5. Hu, H. L. & Chen, L. Q. Three-dimensional computer simulation of ferroelectric domain formation, *J. Am. Ceram. Soc.* **81**, 492-500 (1998).

6. Semenovskaya, S. & Khachaturyan, A.G. Development of ferroelectric mixed states in a random field of static defects, *J. Appl. Phys.* **83**, 5125-5136 (1998).

7. Devonshire, A. F. Theory of barium titanate – part I, *Philos. Mag.* **40**, 1040-1063 (1949).

8. Haun, M. J., Furman, E., Jang, S. J. & Cross, L. E. Thermodynamic theory of the lead zirconate-titanate solid solution system, part I: phenomenology, Ferroelectrics **99**, 13-25 (1989).

9. Fett, T. & Munz, D. Measurement of Young's moduli for lead zirconate titanate (PZT) ceramics, J. Testing Evaluation **28**, 27-35 (2000).
